# 

**Published:** 1917-03-03

**Authors:** 


### The Book Market.

LIST OF NEW BOOKS RECEIVED FROM THE FOLLOWING PUBLISHERS:—

Bale, Son and Danielsson: "Hints for Hospital Orderlies." 6d. net.

Chatto and Windus: "Fry's London Charities." 1s. 6d. net.

H. K. Lewis and Co., Ltd.: "Glaucoma." By R. H. Elliot, M.D. 3s. 6d. net.

Rates of Subscription: Twelve months, 6/6; Six months, 4/-; Three months, 2/-; post free to any add.ess in the United Kingdom. Abroad, Twelve months, 8/8: Six months, 5/-; Three months, 2/6, post free. The Hospital "Index" to Volume LX., April to September 1916, is now ready, price 2½d. per copy, post free. Address The Manager, "The Hospital," The Hospital Building, 28/29 Southampton Street, Strand, London, W.C.

